# Creating Consensus in the Definition of Spinopelvic Mobility

**DOI:** 10.5435/JAAOSGlobal-D-22-00290

**Published:** 2023-06-08

**Authors:** Daniel B. Buchalter, Ashley M. Gall, Aaron J. Buckland, Ran Schwarzkopf, Morteza Meftah, Matthew S. Hepinstall

**Affiliations:** From the Department of Orthopedic Surgery, NYU Langone Health, New York, NY (Dr. Buchalter, Ms. Gall, Dr. Buckland, Dr. Schwarzkopf, Dr. Meftah, and Dr. Hepinstall); and the Melbourne Orthopaedic Group, Spine and Scoliosis Research Associates, Melbourne, Australia (Dr. Buckland).

## Abstract

The term “spinopelvic mobility” is most often applied to motion within the spinopelvic segment. It has also been used to describe changes in pelvic tilt between various functional positions, which is influenced by motion at the hip, knee, ankle and spinopelvic segment. In the interest of establishing a consistent language for spinopelvic mobility, we sought to clarify and simplify its definition to create consensus, improve communication, and increase consistency with research into the hip-spine relationship.

**Methods:**

A literature search was performed using the Medline (PubMed) library to identify all existing articles pertaining to spinopelvic mobility. We reported on the varying definitions of spinopelvic mobility including how different radiographic imaging techniques are used to define mobility.

**Results:**

The search term “spinopelvic mobility” returned a total of 72 articles. The frequency and context for the varying definitions of mobility were reported. 41 papers used standing and upright relaxed-seated radiographs without the use of extreme positioning, and 17 papers discussed the use of extreme positioning to define spinopelvic mobility.

**Discussion:**

Our review suggests that the definitions of spinopelvic mobility is not consistent in the majority of published literature. We suggest descriptions of spinopelvic mobility independently consider spinal motion, hip motion, and pelvic position, while recognizing and describing their interdependence.

Prosthetic hip dislocation remains one of the most vexing problems faced by arthroplasty surgeons today. Until the past decade, efforts to prevent dislocation focused primarily on restoring limb length, offset, and achieving acetabular implant position within safe zones as defined by Lewinnek et al and Callanan et al.^1,2^ Ranawat and others encouraged surgeons to consider the combined effects of femoral and acetabular anteversion, but femoral anteversion being difficult to measure radiographically, this aspect of hip stability remains underexplored.^3,4,5,6^ Dorr^7^ raised awareness of the idea that spinal stiffness and deformity could result in abnormal functional acetabular implant positioning, but the effect of spinal stiffness on dislocation risk was not widely appreciated and little formal inquiry was devoted to spinopelvic parameters.

Several influential studies have demonstrated that abnormal spinal mobility markedly increases the risk of prosthetic hip dislocation.^8-10^ The hip-spine relationship and the influence of spinopelvic mobility on the risk of prosthetic hip dislocation have subsequently emerged as important areas for inquiry.^11^ As expected with an evolving field, published literature on hip-spine issues and spinopelvic mobility contains conflicting and confusing terminology. The American Academy of Orthopaedic Surgeons formed the Hip-Spine Workgroup to help introduce clarity to this complicated field, resulting in the publication of a systemic approach to the hip-spine relationship in 2019.^12^ And while independent investigators exploring this emerging topic have further advanced our knowledge, they continue to introduce new sources of confusion, prompting the present investigation.

The term spinopelvic mobility has most often been applied to motion within the spinopelvic segment. Alternatively, the authors have recently used this term to describe change in pelvic tilt between various functional positions, which is influenced by motion at the hip, knee, and ankle joints in addition to motion within the spinopelvic segment. Those who use the former definition describe patients with stiff spines, either from arthritis or fusion, as having spinopelvic stiffness or hypomobility.^13,14,15,16,17,18,19,20,21,22,23,24,25,26,27,28,29,30^ Those who use the latter definition have sometimes described these patients as having spinopelvic hypermobility.^31,32,33,34^ This discrepancy in definitions when describing the same pathology risks confusion and contradictory recommendations regarding the alterations in the surgical cup positioning target.

This conflicting terminology may relate to competing techniques used to evaluate the hip-spine relationship using sitting and standing radiographs to assess the functional positions of the spine, pelvis, and proximal femur. Although variations exist, there are two distinct approaches commonly used. Most frequently, standing and upright or relaxed-seated radiographs are used.^13,14,15,16,17,18,19,20,22,23,24,25,33,34,35,36,37,38,39,40,41,42,43,44,45,46^ Alternatively, step-up and forward-leaning or flexed-seated radiographs demonstrate the relationship between the femur and the pelvis in at-risk activities, evaluating extreme hip extension with posterior pelvic tilt and extreme hip flexion with anterior pelvic tilt, respectively.^12,21,31,32,47,48,49,50,51,52,53,54,55,56,57,58,59,60^ Radiographs with extreme positioning are well suited to reveal impingement and dislocation risk; however, such positioning can underestimate or reverse the direction of pelvic motion compared with relaxed positioning.^34,48,51-53^ Isolated measurement of pelvic position on flexed-seated radiographs may not reliably reflect spinal stiffness, but flexed-seated radiographs help identify hip users^61^ who achieve functional positions with greater than typical hip motion^61,62^ in the setting of a stiff spine.

In the interests of seeking consensus to enable a consistent language for describing spinopelvic mobility, we sought to determine the frequency and context of use for each definition of spinopelvic mobility, focusing on whether motion was measured within the spinopelvic segment or of the pelvis relative to a vertical axis, and how authors defined spinopelvic hypomobility/hypermobility. We critically examined the published literature and investigated the association between the imaging technique and the definition of spinopelvic mobility used. We then sought to clarify and simplify the definition of spinopelvic mobility to create consensus, improve communication, and increase consistency with research into the hip-spine relationship.

## Methods

A literature search was done using the MEDLINE (PubMed) library to identify all existing articles pertaining to spinopelvic mobility. The search term spinopelvic mobility returned a total of 134 publications. Abstracts were evaluated to find those associated with total hip arthroplasty (THA) procedures. All article types, including clinical trials, case series, case reports, review articles, and published commentaries, were considered. Inclusion criteria encompassed all articles available in full text and all article types. Articles were excluded if they were not published in English, not available in full text, or not related to THA. While assessing relevant studies, two additional articles were discovered based on reviewing the references in the publications identified by the original search. The text and images of included publications were carefully reviewed to determine whether the term spinopelvic mobility was used to describe (1) motion within the lumbosacral spine that affects pelvic position or (2) motion of the pelvis relative to the vertical plane. Furthermore, articles were reviewed to identify any discussion of relaxed-seated radiographs, extreme lateral radiograph positioning, or spinopelvic hypomobility/hypermobility.

## Results

A total of 72 of 136 articles identified fit the inclusion criteria. Of the 64 articles that were excluded, 57 articles were unrelated to THA, six articles were not available in full text, and one article was not written in English. Of the 72 articles included, there were 15 prospective case series, 14 retrospective case series, 27 reviews, 1 clinical trial, 2 prospective diagnostic cohort studies, 1 cross-sectional study, 7 case-control studies, 2 comparative studies, 2 commentaries, and 1 case report.

The term hypermobility was used in 22 articles (11 spinopelvic hypermobility, seven pelvic hypermobility, and four hip-spine hypermobility).^13,14,15,16,17,18,19,20,21,22,23,24,25,26,27,28,29,30,31,32,33,34^ A stiff spine was described as diminished mobility in 18 of these 22 articles.^13,14,15,16,17,18,19,20,21,22,23,24,25,26,27,28,29,30^ Diminished mobility was defined as a change in pelvic tilt or sacral slope from standing to sitting of less than 20° within four of the 18 articles and less than 10° within 14 of the 18 articles (Table 1). Four articles described patients with stiff spines as having spinopelvic hypermobility.^31,32,33,34^ This included a study by Grammatopoulos et al,^31^ which compared lateral-standing radiographs to flexed-seated radiographs and defined spinopelvic hypermobility as a change in pelvic tilt >30° between the two radiographs. The other three of these articles (two case series^33,34^ and one literature review^32^) cite Grammatopoulos et al to reiterate that patients with spinal fusions and spinopelvic hypermobility are at higher risk of instability and poor outcome.

**Table 1:** Published Definitions of Spinopelvic Hypermobility

Authors	Semantic Definition of Hypermobility	Numeric Definition of Hypermobility	Numeric Definition of Stiffness	Radiographs Used to Determine Mobility		Hypermobility Used to Describe
				Standing, Upright Seated	Standing, Flexed/Deep Seated	
Attenello and Harpstrite^27^	Hypermobile pelvis	>30° ∆SS	<10° ∆SS	✓		Flexible spine
Eftekhary et al^15^	Spinopelvic hypermobility	∆PT > 30°	∆PT < 20°	✓		Flexible spine
Grammatopoulos et al^31^	Spinopelvic hypermobility	∆PT > ±30°	∆PT< ±10°		✓	Stiff spine resulting in increased hip motion
Haffer et al^33^	Spinopelvic hypermobility	>30° ∆PT	∆PT < 10°	✓		Stiff spine resulting in increased hip motion
Haffer et al^26^	Hypermobile pelvis	>30° ∆SS	<10° ∆SS	✓		Flexible spine
Heckmann et al^17^	Spinopelvic hypermobility	>30°∆SS	<10°∆SS	✓		Flexible spine
Heckmann et al^24^	Pelvic hypermobility	>30° ∆SS	<10° ∆SS	✓		Flexible spine
Ike et al^25^	Hip-spine hypermobility	>30° ∆SS	< ±10° ∆SS	✓		Flexible spine
Innmann et al^34^	Spinopelvic hypermobility	∆PT > ±30°	∆PT < ±10°	✓		Stiff spine resulting in increased hip motion
Innmann et al^22^	Hip-spine hypermobility	∆PT > 30°	∆PT < 10°	✓		Flexible spine
Innmann et al^21^	Pelvic hypermobility	∆PT > 30°	∆PT < 10°	✓		Flexible spine
Innmann et al^32^	Spinopelvic hypermobility	∆PT > 30°	∆PT < 10°		✓	Stiff spine resulting in increased hip motion
Kanawade et al^13^	Pelvic hypermobility	Posterior tilt >35°	Posterior tilt <20°	✓		Flexible spine
Lee et al^19^	Spinopelvic hypermobility	>30° ∆SS	<10° ∆SS	✓		Flexible spine
López et al^20^	Spinopelvic hypermobility	>40° ∆SS	<20° ∆SS	✓		Flexible spine
Lum et al^18^	Spinopelvic hypermobility	∆ST > 30°	∆ST < 10°	✓		Flexible spine
Lum et al^28^	Hypermobile pelvis	∆ST > 35°	∆ST < 20°	✓		Flexible spine
Mancino et al^29^	Hip-spine hypermobility	>30° ∆SS	<10° ∆SS	✓		Flexible spine
McKnight et al^16^	Spinopelvic hypermobility	>30° ∆SS	<10° ∆SS	✓		Flexible spine
Nikkel et al^30^	Spinopelvic hypermobility	>30° ∆SS	<10° ∆SS	✓		Flexible spine
Stefl et al^14^	Hip-spine hypermobility	∆ST > 30°	∆ST < 10°	✓		Flexible spine
Watanabe et al^23^	Pelvic hypermobility	>30° ∆SS	<10° ∆SS	✓		Flexible spine

Radiographic assessment with extreme positioning was used or discussed within 17 of the 72 relevant spinopelvic mobility articles (Table 2).^12,21,31,32,47,48,49,50,51,52,54,55,56,57,58,59,60^ Only three of these 17 used the term hypermobility, and two of these three were referring to a stiff spine. Of the 41 articles that used standing and upright or relaxed-seated radiographs without the use of extreme positioning, 19 used the term hypermobility and only two of these were referring to a stiff spine. Of the eight articles that used both standing to upright relaxed-seated and extreme positioning radiographs, two used the term hypermobility and one of these was referring to a stiff spine.

**Table 2:** Extreme Positioning Radiographs Used in the Evaluation of Spinopelvic Mobility

Authors	Relaxed Seated	Flexed/Deep Seated	Standing	Step-Up	Standing with Maximum Anterior Pelvic Rotation and Maximum Posterior Pelvic Rotation	Swing Phase and Stance Phase	Standing Pivot and Turn Poses	Squatting
Attenello and Harpstrite^27^	✓		✓					
**Behery et al** **^51^**	✓	✓	✓	✓				
Berliner et al^44^	✓		✓					
**Bracey et al** **^54^**		✓	✓	✓				
Buckland et al^39^	✓		✓					
Buckland et al^40^	✓		✓					
Buckland et al^9^	✓		✓					
**Buckland et al** **^52^**		✓	✓	✓				
Carender et al^38^	✓		✓					
De Leon et al^63^	✓		✓					
Eftekhary et al^15^	✓		✓					
**Eftekhary et al** **^12^**	✓	✓	✓					
**Elbuluk et al** **^57^**	✓	✓	✓					
Esposito et al^41^	✓		✓					
Frandsen et al^64^	✓		✓					
**Grammatopoulos et al** **^31^**		✓	✓					
**Gu et al** **^58^**		✓	✓					
Haffer et al^33^	✓		✓					
Haffer et al^26^	✓		✓					
**Hayden et al** **^50^**			✓		✓			
Heckmann et al^17^	✓		✓					
Heckmann et al^24^	✓		✓					
Heckmann et al^35^	✓		✓					
Homma et al^36^	✓		✓					
**Innmann et al** **^21^**	✓	✓	✓					
Innmann et al^43^	✓		✓					
Innmann et al^22^	✓		✓					
Innmann et al^34^	✓		✓					
**Innmann et al** **^32^**	✓	✓	✓					
Ike et al^25^	✓		✓					
Kanawade et al^13^	✓		✓					
**Klemt et al** **^56^**			✓			✓		
Langer et al^65^	✓		✓					
Lazennec et al^8^	✓		✓					
Lee et al^19^	✓		✓					
López et al^20^	✓		✓					
Lum et al^28^	✓		✓					
Lum et al^18^	✓		✓					
**Luthringer et al** **^47^**	✓	✓		✓				
Mancino et al^29^	✓		✓					
**McCarthy et al** **^60^**		✓	✓				✓	
McKnight et al^16^	✓		✓					
Niemeier et al^66^	✓		✓					
Nikkel et al^30^	✓		✓					
Ochi et al^42^	✓		✓					
Onggo et al^67^	✓		✓					
Padgett^68^	✓		✓					
Perticarini et al^69^	✓		✓					
**Ransone et al** **^48^**	✓	✓	✓					
**Rivière et al** **^59^**	✓		✓					✓
Sharma et al^70^	✓		✓					
Shen et al^46^	✓		✓					
Stefl et al^14^	✓		✓					
Tezuka et al^37^	✓		✓					
**Vigdorchik et al** **^55^**		✓	✓					
**Vigdorchik et al** **^49^**		✓	✓					
Vigdorchik et al^45^	✓		✓					
Watanabe et al^23^	✓		✓					

Bold denotes authors who used extreme positioning.

## Discussion

Grammatopoulos et al^31^ received the 2018 Frank Stinchfield Award when they reported that THA patients with spinal arthrodesis who have spinopelvic hypermobility have inferior outcomes and higher rates of hip instability. Spinopelvic mobility was classified based on standing and flexed-seated lateral radiographs as normal if pelvic tilt changed ±10° to 30°, stiff <±10°, or hypermobile >±30°. Importantly, these definitions were previously formulated based on upright-sitting radiographs, not flexed-seated radiographs that were being used in their study. In addition, the authors of this study explicitly acknowledged that their definition of spinopelvic mobility referenced movement of the pelvis relative to a vertical plane, not movement within the lumbosacral spine. They reported that 28.5% of THA patients with spinal arthrodesis have spinopelvic hypermobility, all of which demonstrated anterior tilting of the pelvis during flexed sitting. Importantly, although it is plausible that a person with a stiff spine may have a change in sacral slope or pelvic tilt <10° when transitioning from standing to relaxed-seated and a change in sacral slope or pelvic tilt >30° when transitioning from standing to flexed-seated, this same patient cannot reasonably be classified as having both spinopelvic hypomobility and spinopelvic hypermobility. Conversely, Inman et al^21^ reported spinopelvic hypermobility in 19% of patients with severe hip osteoarthritis (OA), compared with 2% of control subjects; these patients with stiff hips may be spine users rather than having truly hypermobile spines. The terminology used by Grammatopoulos et al is important because their study has notable merit, deservedly received the 2018 Frank Stinchfield award, and was highlighted in 2021 as one of the knowledge resources for the American Board of Orthopaedic Surgery's Web-Based Longitudinal Assessment. Importantly, however, this terminology contradicts our intuitive understanding, along with the bulk of the published literature.

The presence of contradictory definitions of spinopelvic mobility in the published literature is one reason that discussions of the hip-spine relationship remain confusing. From our findings and these contradictory definitions, it is clear that flexed-seated and relaxed-seated radiographs should be analyzed, interpreted, and discussed differently. To minimize confusion, we specifically suggest that the phrase spinopelvic hypermobility be discarded and more specific terms separating spine and hip mobility be used.

Importantly, there is general agreement that the following relationship prevails: patients with stiff spines require additional hip, knee, and even ankle motion to accomplish sitting and standing, whereas patients with a stiff pelvis/hip require additional lumbosacral range of motion to accomplish the same activities.^32,34^ Competing language used to describe this consensus should not confuse or distract from this core understanding.

### Understanding Flexed-Seated Analysis

**Figure 1:**
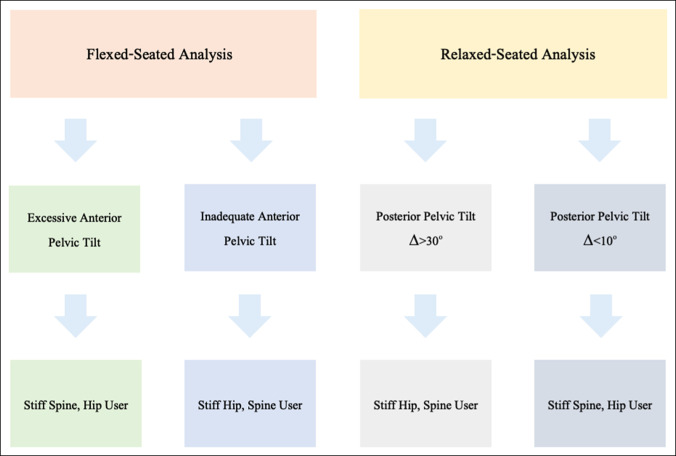
Chart showing hip versus spine users according to flexed-seated versus relaxed-seated analysis. Algorithm to define hip users and spine users according to flexed-seated versus relaxed-seated analysis.

Flexed-seated analysis is an active process used to replicate a patient transferring their weight forward as they get out of a chair. This position helps identify spine users and hip users. Those patients who have greater changes in lumbar lordosis in this position require less anterior pelvic tilt to move their gravity line anterior to their feet to initiate standing from a seated position and thus can be considered spine users. Those patients who have greater increase in anterior pelvic tilt in this position do so because they have lumbosacral spinal stiffness and thus can be considered hip users with spinal hypomobility (Figure 1).^51^

Buckland et al^52^ recognized this phenomenon and reported that patients with degenerative flatback deformities had greater pelvic motion (measured by Δ change in pelvic tilt) rather than spinal motion (measured by Δ change in lumbar lordosis) with postural changes as measured using supine, standing, flexed-seated, and stepping-up lateral radiographs. In a series of 288 patients, they reported that patients with flatter backs required more postural pelvic tilt to compensate for smaller changes in lumbar lordosis from standing, sitting, and supine positions (*P* < 0.001). Thus, Buckland et al clarified that greater pelvic tilt was not a matter of spinopelvic hypermobility but rather a compensatory change where the pelvis follows the trunk because of relative spinal hypomobility.

### Understanding Relaxed-Seated Analysis

Relaxed-seated analysis is a passive process used to replicate the simultaneous relaxation of the lumbar spine and subsequent accompanying posterior pelvic tilt that occurs when sitting. Therefore, in relaxed-seated analysis, patients with lumbosacral hypomobility (stiff spines), defined as <10° to 20° change in pelvic tilt or sacral slope from standing to sitting,^13,14,15,16,17,18,19,20,21,22,23,24,25,26,27,28,29,30^ have limited passive relaxation of their lumbar lordosis, which subsequently drives a limited posterior pelvic tilt. Buckland et al^40^ found lumbar spinal degeneration to exist on a spectrum with the most severe spinal degeneration being associated with the greatest reduction in posterior pelvic tilt during relaxed sitting. Heckmann et al explained that decreased pelvic motion from degenerative lumbar stiffness is accompanied by increased femoral motion to allow for changes in posture.^53^ These patients are again considered hip users.^35,41,62,71^ Specifically, for every 1 degree of loss of pelvic mobility, femoral mobility must increase by 1° to allow for an upright position during sitting.^35,62^

Alternatively, greater-than-usual change in posterior pelvic tilt with relaxed-seated analysis occurs in response to reduced hip range of motion—patients with stiff hips are spine users. Using severe hip osteoarthritis as a surrogate for hip stiffness, Buckland et al^39^ found that those patients with severe hip osteoarthritis had twice as large a change in posterior pelvic tilt when going from standing to relaxed sitting (*P* < 0.001). They concluded that when patients are unable to flex their hips to sit in a chair because of degenerative changes causing capsular contractures and pain, they require greater posterior pelvic tilt to achieve a seated position. Similarly, patients with hip flexion contractures may require anterior pelvic tilt to stand. In a study looking at 136 patients with this type of pelvic hypermobility defined as a change in sacral slope from standing to sitting ≥30°, Sculco et al^72^ found that pelvic hypermobility resolved in 95% of these patients after THA. This supports the notion that hip stiffness requires greater change in pelvic tilt to achieve sitting and standing positions; once hip motion improves, apparent pelvic hypermobility is alleviated (Figure 1).

Although this phenomenon has been called spinopelvic hypermobility, it is unlikely to reflect an increase in the available range of motion within the spinopelvic segment, which includes the lumbosacral spine and the pelvis. It rather reflects the requirement to use an increased portion of the available motion to accomplish basic tasks. A stiff spine demands that the pelvis tilt anteriorly to achieve flexed sitting, resulting in an apparent increase in the motion of (but not within) the entire spinopelvic segment when flexed-seated imaging is used. Because this motion occurs at the hip, not within the lumbosacral spine, calling this phenomenon spinopelvic hypermobility is confusing. Of the articles discovered during our literature review, 21 discussed hypermobility in the setting of relaxed-seated positioning. Of these 21 articles, seven clearly discussed pelvic hypermobility as compensating for stiffness in the hips. The remaining articles spoke for either hip-spine hypermobility or spinopelvic hypermobility and did not clearly explain that apparent hypermobility was often a compensation for hypomobility in an adjacent body segment.

## Conclusion

Several authors have adopted the term spinopelvic hypermobility in the setting of spinal fusion. Our review suggests that such terminology is not consistent with most of the published literature. Hence, rather than using the term spinopelvic hypermobility, we advocate adopting the terminology of Inman et al,^61^ who describe these patients with stiff spines as being hip users.

Moving forward, we suggest descriptions of spinopelvic mobility independently consider spinal motion, hip motion, and pelvic position, while recognizing and describing their interdependence. Stiff spines require patients to become hip users, stiff hips create spine users, and patients with combined spinal and hip stiffness experience severe disability because they are unable to compensate in one place for stiffness in another. Although both relaxed-seated analysis and flexed-seated analysis can independently predict a hip user or a spine user, it is important that future studies define different normative parameters for flexed-seated analysis rather than extrapolating those created for relaxed-seated analysis (normal pelvic tilt change of ±10° to 30°, stiff <±10°, or hypermobile >±30°).
